# Shone᾽s complex in pediatric age group: clinical characteristics, structural components, interventions, and outcomes of a cohort from a single center

**DOI:** 10.1186/s12887-025-05716-3

**Published:** 2025-05-16

**Authors:** Shaimaa Rakha, Marwa Said Ali, Ahmad Abd El Aleem El Derie, Yahya Wahba, Mohammed Magdy Abo Elkheir

**Affiliations:** 1 Pediatric Cardiology Unit, Department of Pediatrics, Faculty of Medicine, Mansoura University, Mansoura, Egypt; 2 Mansoura University Children’s Hospital, Mansoura, Egypt; 3 Pediatric Cardiac Surgery Unit, Cardiac Surgery Department, Faculty of Medicine, Mansoura University, Mansoura, Egypt; 4 Genetics Unit, Department of Pediatrics, Faculty of Medicine, Mansoura University, Mansoura, Egypt

**Keywords:** Characteristics, Shone, Complex, Pediatric

## Abstract

**Background:**

Shone’s complex (SC) comprises multi-level left-sided obstructive cardiac lesions. Limited research has addressed SC in the pediatric age, especially the factors impacting outcomes. Therefore, the current work aimed to study clinical, structural, interventional aspects, and outcomes of SC cohort in childhood.

**Methods:**

A retrospective analysis of SC cases from a single tertiary center was conducted. Demographics and clinical data were collected. Echocardiographic data, other imaging modalities, and required interventions (surgical or transcatheter) were determined. Outcomes regarding morbidity and mortality were collected.

**Results:**

Twenty five cases were included;17 (68%) were males with a median age of one month (0.2–4.8) at presentation and 33 (5–60) months at the last follow-up. All patients were diagnosed with incomplete SC, with the commonest lesions: Parachute mitral in 86% and aortic coarctation (COA) in 72%. Cardiac multi-slice CT (MSCT) was indicated in 16 (64%), and transesophageal echocardiography in 2 (8%). No intervention was performed in 5 (20%) cases, while interventional catheterization was performed in 4 (16%) and surgery was performed in 19 (76%). The mortality rate in the cohort was 3 (12%), with age of the patient, weight, and weight Z-score at the last follow-up significantly lower in deceased cases. The proportion of cases with suppressed myocardial contractility at the initial presentation was statistically significant in the deceased group (*p* = 0.03). However, anthropometrics and demographics at presentation, SC components, and interventions did not differ significantly between the two groups.

**Conclusion:**

Shone’s complex is primarily presented in the atypical incomplete subtype. In pediatric age, parachute mitral and COA are the most common lesions. Echocardiography with MSCT could achieve a non-invasive, comprehensive diagnosis and guide the required intervention. The family should be informed of potential recurrence, progression, or reintervention for SC lesions, emphasizing the need for prolonged follow-up.

## Introduction

Congenital heart disease (CHD) is the most common major congenital malformation, with a birth prevalence of 9.4 per 1000 live births [1]. Shone's complex (SC) is a combination of multi-level left-sided obstructive cardiac lesions first described in 1963 by Dr. John D. Shone [2]. The prevalence of this rare constellation of congenital abnormalities is approximately 0.7% of all CHD [3].

Shone et al. initially divided the complex into typical (complete) and atypical (incomplete). He described complete Shone's complex as a category consisting of left-sided obstruction at four levels: supramitral membrane, valvular mitral stenosis (MS) due to parachute mitral valve (PMV), subaortic stenosis, and coarctation of the aorta (COA). When two or three levels of obstruction are present, it is referred to as atypical SC [2]. Other obstructive lesions have been suggested to be added to the complex combinations to reach six lesions, as adopted by the International Pediatric and Congenital Cardiac Code of 2021 [4], or up to eight lesions, as described by the Adult Congenital Heart Association [5]. The proposed additional left obstructive lesions to be part of the complex include cor triatriatum, bicuspid aortic valve (BAV), small aortic valve annulus, hypoplastic (stiff) left ventricle, and small aortic arch [5].

The complex's multi-level left-sided lesions cause inflow and outflow obstruction of the left ventricle (LV) of variable severity from mild to severe. The etiologic background is not entirely clear; however, mitral valve obstruction has been suggested as the initial pathological event in SC during early embryogenesis, resulting in underdevelopment of the LV cavity, giving rise to various degrees of LV outflow tract obstruction and COA [6]. Nevertheless, this is only partially true, as in some patients, the LV outflow and descending aorta develop obstruction without inflow stenosis and vice versa.

This anomaly is traditionally reported in infants and children and rarely in young adults, where the clinical presentation and post-surgical prognosis may have distinctive features [7, 8]. It is highly unusual for a patient to remain utterly asymptomatic throughout childhood and be incidentally detected during adulthood [9].

Limited research has addressed the characteristics of pediatric patients with SC, particularly the factors that impact the outcome of pediatric cases. Therefore, the current work aimed to characterize the clinical aspects, echocardiographic features, non-invasive imaging required, and outcome determination regarding mortalities and morbidities in pediatric patients with SC.

## Material and methods

The retrospective observational cross-sectional study included patients diagnosed with Shone's complex at Mansoura University Children's Hospital in Egypt between June 2014 and June 2022. The Institutional Review Board (IRB) of Mansoura University, Faculty of Medicine, Egypt, approved the study; approval code number MS.21.07.1594. For all study subjects, informed consent was obtained from patients'guardians to use anonymous data. The study included pediatric patients under 18 years of age with a confirmed diagnosis of the complex, defined as the presence of at least two left-sided cardiac obstructive lesions diagnosed by echocardiography or other imaging techniques, in accordance with the primary diagnostic lesions described by Shone et al. [2]. Patients with functionally univentricular heart physiology were excluded from the study.

### Demographic and clinical data

The demographics collected included sex and age at the initial diagnosis and last follow-up. Data on the primary presenting symptoms, required medical treatments, and family history of CHD were also collected.

### Transthoracic echocardiography (TTE)

Standard detailed transthoracic echocardiograms were performed in accordance with the recommendations of the American Society of Echocardiography [10]. The following echocardiographic modalities were used:

1. 2D echocardiography with color flow: For a) Determination of number, position, morphology, and severity of left-sided obstructive, b) Secondary changes such as left atrial dilatation, left ventricular hypertrophy, and pulmonary hypertension, and c) Associated cardiac anomalies.
2. Pulsed and continuous wave Doppler: including flow velocities and pressure gradients across the mitral valve, aortic valve, left ventricular outflow, and descending aorta.
3. M-mode: LV systolic function was estimated using fractional shortening (FS) and ejection fraction (EF).

### Other imaging modalities

Other utilized diagnostic imaging modalities were documented, such as fetal echocardiography, transesophageal echocardiography (TEE), and cardiac multi-slice CT angiography (MSCT).

### Interventions

Required interventional management was documented, whether catheter-based interventions or surgical operations. Transcatheter interventions were performed in accordance with the scientific statement from the American Heart Association [11]. For surgical repair, the decision was determined through a multidisciplinary meeting between pediatric cardiology and cardiac surgery depending on the severity of obstruction determined by pressure gradients across the obstruction or Z-score (for subaortic diameter, valves, or aortic arch) with relevance to the clinical status of the patient [12, 13].

### Outcome

Investigated outcomes included mortality and morbidities such as hospital admissions or persistent systemic hypertension. Based on mortality, the included cohort was subdivided into two groups: alive and deceased. The two groups were compared to detect differences in clinical data, SC components, and interventions.

### Statistical analysis

The statistical analyses were conducted using SPSS software (version 25, IBM Corp., Armonk, NY, USA). Qualitative data were described using numbers and percentages. Quantitative data were presented as median (IQR) for non-normally distributed data and as mean ± standard deviation for normally distributed data after testing normality using the Shapiro–Wilk test. Fisher's exact test was used to compare qualitative data between groups. The Mann–Whitney U test was used to compare the two studied groups for non-normally distributed data. The obtained results were considered statistically significant when the *P*-value was ≤ 0.05. The Kaplan–Meier survival curve was used to plot the overall survival in relation to interventions.

## Results

**Fig. 1:**
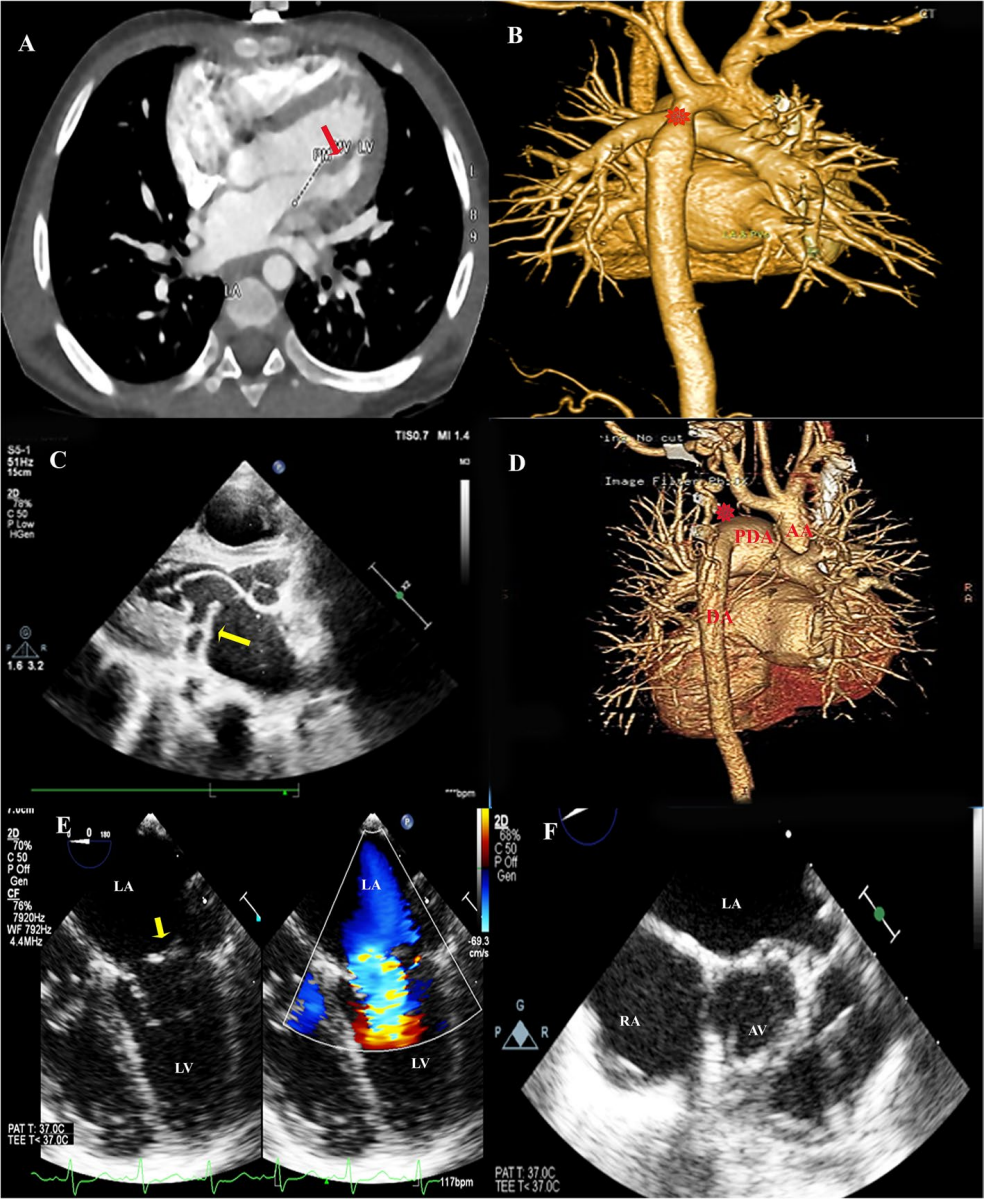
Examples of Diagnostic imaging for some of the SC cases requiring surgical intervention. A, B) axial CT cut showing dilated LV& LA with parachute mitral valve (red arrow) and 3D volume rendering demonstrating COA in the same patient (red asterisk). C, D) TTE of long parasternal view demonstrating supramitral membrane and 3D volume rendering CT showing interrupted aortic arch in the same patient (red asterisk at the interruption). E, F) TEE of four-chamber view demonstrating turbulent flow across supramitral membrane (yellow arrow) with parachute mitral and short axis view showing BAV in the F panel. AA: ascending aorta, AV: aortic valve, DA: descending aorta, LA: left atrium, LV: left ventricle, PDA: patent ductus arteriosus, and RA: right atrium

Table 1 shows the demographic and clinical characteristics of the included patients. The study included 25 patients, predominantly males, with a median age of one month at the initial presentation. Heart failure symptoms were the most common presentation in 52% of cases, followed by subjective cyanosis. The family history of CHD was positive for 16% of the cohort. In two patients, sibling death was linked to major CHD: hypoplastic left heart syndrome in one case and an unknown complex CHD in the other. Another patient had a grandfather with ASD, and the other had a cousin with valvular aortic stenosis. All cases were diagnosed using TTE, with two patients requiring TEE for further mitral/supra mitral evaluation, and 16 (64%) had cardiac MSCT for great arteries evaluation. Figure 1 demonstrates examples of diagnostic imaging for SC cases that required surgical intervention.

**Table 1:** Patient demographics and clinical characteristics

**Variable**		Results^*^
Age	*At presentation (months)*	1 (0.2–4.8)
	*At last follow-up (months)*	33 (5–60)
Sex (Male)		17 (68%)
Weight	*at presentation (kg)*	3.8 (2.9–5.7)
	*Z-score at presentation*	−1.4 (−2.1, −0.4)
	*Weight at last follow-up (kg)*	11.5 (5.8–15.5)
	*Weight Z-score at last follow-up*	−0.8 (−1.6, −0.03)
Height	*at presentation (cm)*	56.5 ±12.6
	*Z-score at presentation*	−0.4 (−1.0, 0)
	*at last follow-up (cm)*	87 (62–100.5)
	*Z-score at last follow-up*	−0.4 (−1.1, 0.4)
Presenting Manifestation	*Heart Failure Symptoms*	13 (52%)
	*Cyanosis*	5 (20%)
	*Failure to Thrive*	1 (4%)
	*Chest Infection*	1 (4%)
	*Murmur*	3 (12%)
	*Hypertension*	1 (4%)
	*Intrauterine diagnosis*	1 (4%)
Medication	*Diuretic*	15 (60%)
	*Beta Blocker*	2 (8%)
	*Inotrope*	4 (16%)
	*Prostaglandin E2*	2 (8%)
	*others*	11 (44%)
Family History of CHD		4 (16%)
Imaging Modality	Fetal Echo	1 (4%)
	TTE	25 (100%)
	TEE	2 (8%)
	Cardiac MSCT	16 (64%)

*MSCT* multi-slice computed tomography, *TEE* transesophageal echocardiography, *TTE* transthoracic echocardiography

^*^Data are presented as mean ± SD or median (interquartile range), or number (%)

Table 2 demonstrates the structural data of the included patients. All the study cases were diagnosed as partial SC type; none showed complete SC type. The most commonly encountered obstructive lesion was a parachute mitral valve in 84% of cases, followed by COA in 72%. Pulmonary hypertension was detected in 4 (16%) patients during infancy; three cases were diagnosed in the neonatal period and one at the age of five months. One patient was diagnosed with a PMV causing severe mitral stenosis and mild mitral regurgitation in association with complex LVOT obstruction; another case presented with severe COA and moderate inflow stenosis due to parachute-like mitral valve associated with subaortic VSD and significant PDA, and the third case had interrupted aortic arch with PMV causing severe stenosis. The last patient had severe aortic coarctation with supramitral ring and parachute mitral valve. Three of those who had pulmonary hypertension regressed postoperatively, and unfortunately, one patient expired before intervention. Moreover, for subaortic stenosis, one case demonstrated complex LVOT stenosis with a subaortic membrane (SAM) and diffuse narrowing of the LVOT; another case had a fibromuscular ridge; however, 11 patients demonstrated a simple SAM. None of the study cases showed supravalvular aortic stenosis.

**Table 2:** Structural data of the included patients with SC

Variable		Results*
Types of SC	Partial SC	25 (100%)
	Complete SC	0 (0%)
Components of SC	Supra-mitral membrane	4 (16%)
	Parachute mitral valve 1. Parachute-like 2. Full parachute	21 (84%) 14/21 (66.7%) 7/21 (33.3%)
	Subaortic stenosis	13 (52%)
	Aortic coarctation 1. Segment 2. Discrete	18 (72%) 11/18 (61.1%) 7/18 (38.9%)
	Others: 1. Bicuspid aortic valve 2. Hypoplastic aortic arch 3. Cortriatriatum sinister	13 (52%) 2 (8%) 1 (4%)
Secondary cardiac changes	LV inflow stenosis	21 (84%)
	Mitral regurgitation	7 (28%)
	LV outflow stenosis	18 (72%)
	Left atrial dilatation	20 (80%)
	Left ventricle hypertrophy	18 (72%)
	Pulmonary hypertension	4 (16%)
Associated CHD *11(44%)*	VSD	10 (40%)
	PDA	5 (20%)
	ASD	2 (8%)
	Interrupted aortic arch (type A & type B)	2 (8%)
	PLSVC	2 (8%)
	Aberrant right subclavian artery	1 (4%)

*ASD* atrial septal defect, *CHD* congenital heart disease, *PDA* patent ductus arteriosus, *PLSVC* persistent left superior vena cava, *SC* Shone’s complex, *VSD* ventricular septal defect

^*﻿^Data are presented as numbers (%)

Table 3 lists the echocardiographic data, including the study cohort's conventional Doppler parameters and M.mode-derived systolic functions. Although the mean value of EF and FS of the cohort was within the average, four cases demonstrated suppressed myocardial contractility at presentation, with FS being less than 28%.

**Table 3:** Echocardiographic data of the study cohort, including Doppler and M.mode-derived systolic functions

Variable		Results*
Mitral valve	Z-score (*n*=23)	−0.4 (−1.5-0.2)
	E Wave Vmax (m/s)	1.63 ±0.5
	A Wave Vmax (m/s)	1.68 ±0.5
	Mean PG (mmHg)	8.57 ±4.6
	Mitral regurgitation 1. Mild 2. Moderate	7 (28%) 6 (85.7%) 1 (14.3%)
Aortic valve	Z-score (n=20)	−0.35 (−1.8-0.19)
	Vmax (m/s)	1.92 ±1
	Max PG (mmHg)	10 (6–26)
	Aortic regurgitation (mild)	1 (4%)
Left ventricular outflow (Subaortic)	Vmax (m/s)	1.5 (1–2.6)
	Max PG (mmHg)	9.5 (4.1–25)
Descending aorta	Vmax (m/s)	2.7 ±1.2
	Max PG (mmHg)	34.6 ±9.1
M.mode-derived systolic functions	EF (%)	66.8 ±10.5
	FS (%)	34.2 ±5.5

*EF* Ejection fraction; *FS*: Fractional shortening, *Max* maximum, *PG* Pressure Gradient, *Vmax* Maximum Velocity (m/sec)

^*^Data are presented as mean ± SD or median (interquartile range), or number (%)

Table 4 shows the required transcatheter and surgical interventions for the studied patients. Five catheter interventions were performed in 4 (16%) cases, with one patient having two separate transcatheter interventions: aortic balloon angioplasty for COA and later balloon aortic valvuloplasty for progressive aortic valve stenosis. Surgical interventions were performed for 19 (76%) cases, with more than half of the patients undergoing COA repair with or without arch reconstruction. In 20% of patients, another cardiac surgical maneuver unrelated to primary SC lesions was performed, such as VSD repair or PDA ligation.

**Table 4:** Transcatheter and surgical interventions that were performed for the studied patients

Variable			Result*
Transcatheter Interventions *4 (16%)*	Age at Catheter (months)		19 (5.38–28.5)
	Types	Balloon angioplasty for COA	1 (4%)
		Balloon aortic valvuloplasty	2 (8%)
		Insertion of pacemaker	1 (4%)
		Device closure for post-surgical residual PDA	1 (4%)
Surgical interventions *19 (76%)*	Age at Surgery (months)		3.5 (2.9–14.3)
	Hospital Stay postoperative (days)		7 (6–14)
	Types	COA repair ± arch reconstruction	14 (56%)
		Subaortic membrane resection	4 (16%)
		MV repair with division of parachute MV	3 (12%)
		Others**	5 (20%)

*COA* coarctation of the aorta, *MV* mitral valve

^*^Data are presented as median (interquartile range), or number (%)

^**^PDA ligation, VSD repair, pacemaker, myomectomy

**Fig. 2:**
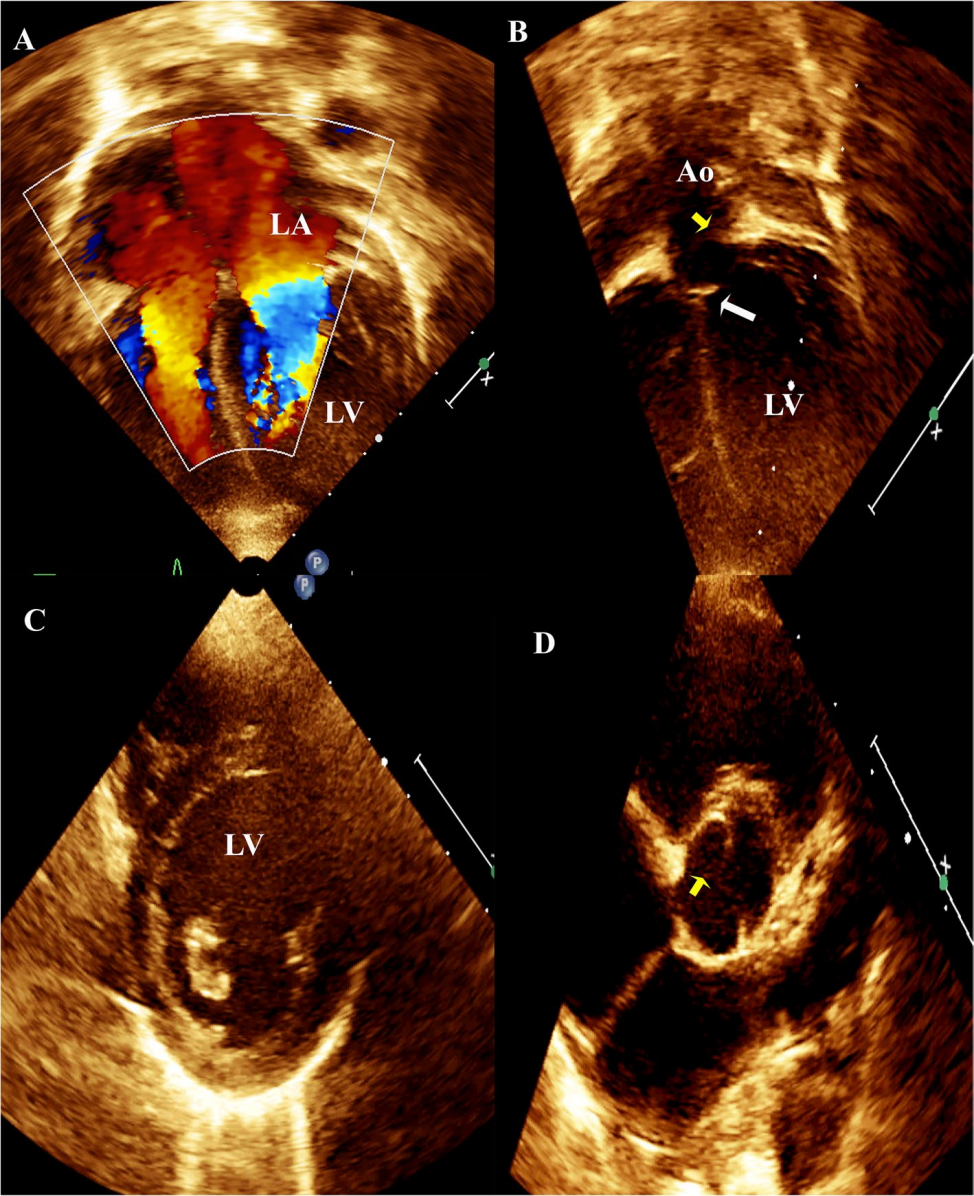
Echocardiography of a 20-month-old boy with incomplete SC requiring no intervention at the time of diagnosis. A) Four-chamber view showing turbulent flow across mitral valve B) Incomplete SAM (white arrow). The yellow arrow points to the aortic valve. C) Short axis at the papillary muscle level demonstrating prominent posterolateral and rudimentary anteromedial papillary muscle (Parachute-like). D) BAV with yellow arrow points to the closure line. Ao: ascending aorta, LA: left atrium, and LV: left ventricle

**Fig. 3:**
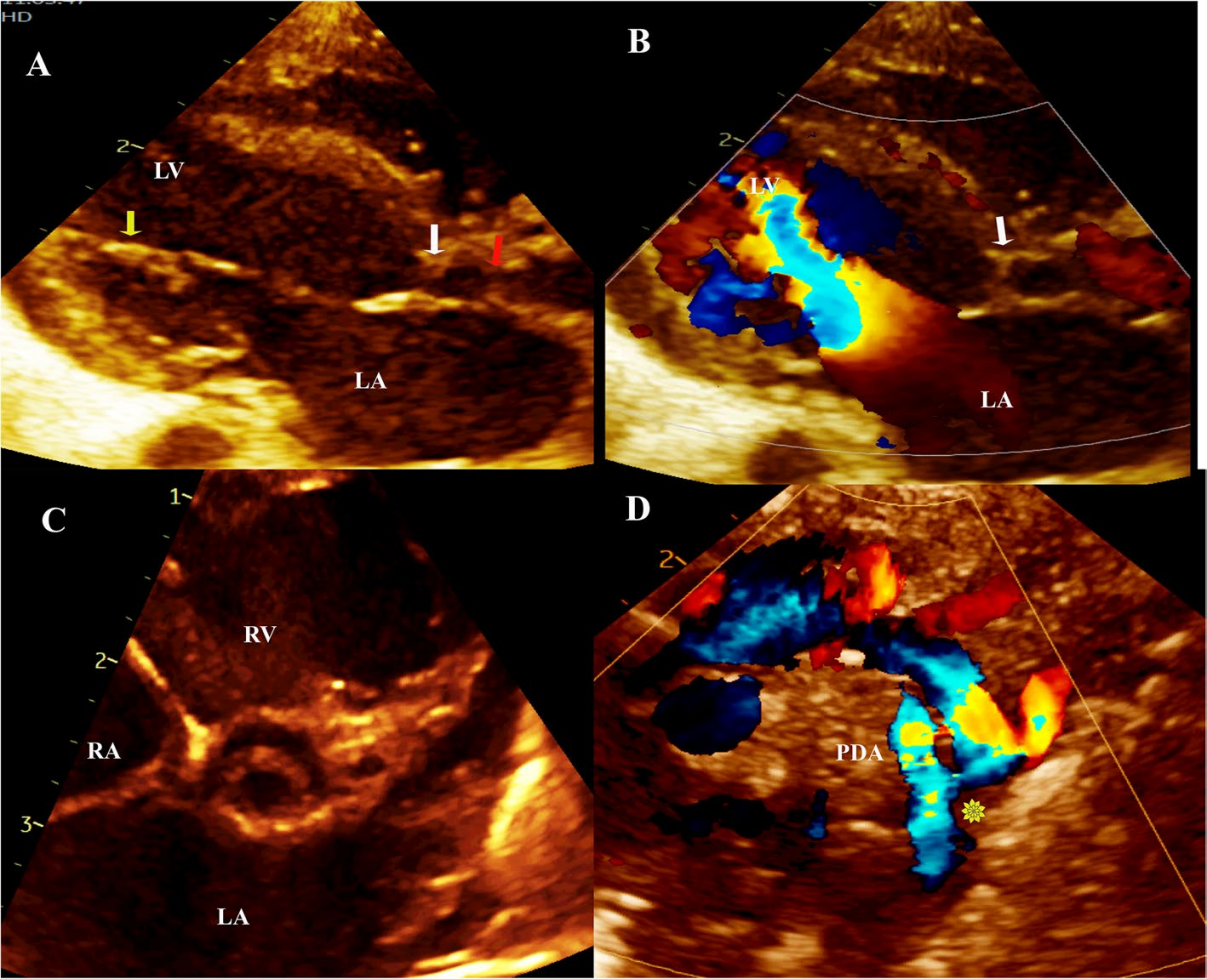
Echocardiography of a newborn with SC who died before a scheduled intervention. A, B) Color-compare of long parasternal view demonstrating turbulent flow across parachute mitral valve (yellow arrow), SAM (white arrow), and thickened aortic valve (red arrow). C) Short axis view showing unicuspid aortic valve. D) Long suprasternal view showing critical COA (yellow asterisk) with PDA-dependent systemic circulation. LA: left atrium, LV: left ventricle, PDA: patent ductus arteriosus, RA: right atrium, and RV: right ventricle

The course of SC during pediatric age, morbidities, and mortality are demonstrated in Table 5. Interventions (transcatheter or surgical) were implemented in 20 (80%) cases. Five cases had no intervention: two patients expired before a scheduled surgery and the other three had mild to moderate obstructive lesions that were non-indicated for interventions. Figure 2 shows echocardiographic images of a 20-month-old boy with incomplete SC requiring no intervention at the time of diagnosis. Figure 3 demonstrates echocardiographic images of a newborn with SC who died before a scheduled intervention. Three (12%) patients had a reintervention due to significant progressive obstruction. One patient who had initial coarctectomy with end-to-end anastomosis at the age of 44 days showed progressive stenosis in a PMV to a severe degree when she underwent a second operation at the age of 15 months for division of fused mitral papillary muscles with mitral valve repair, myomectomy, ASD closure, and pacemaker insertion for heart block. Another case required aortic arch repair at the age of 11 days; had transcatheter aortic valvuloplasty for severe aortic stenosis at 2.5 months, with mild residual stenosis that slowly progressed to moderate at the age of 30 months, and the last case of the reintervention group exhibited severe coarctation with moderate bicuspid aortic valve stenosis when he was two-year-old that required transcatheter balloon angioplasty then balloon valvuloplasty of the aortic valve that progressed to severe at age of 4 years.

**Table 5:** Course of SC, mortality, and morbidity in the studied patients

Variable		N	%
Course of the disease	1. Intervention (Cath and/or Surgery)		
	*Performed	20	80%
	*No (nonindicated/died preintervention)	5	20%
	2. Need reintervention/progressed and performed reintervention	3	12%
	3. Progressed but no reintervention required yet	16	64%
Morbidity *11 (44%)*	**1. Hospital admission**	4	16%
	*For pneumonia	2	8%
	*For decompensated heart failure	2	8%
	**2. Persistent Systemic Hypertension**	7	28%
Mortality *3 (12%)*	1. After intervention	1	4%
	2. Without Intervention	2	8%

^*^Data are presented as numbers (%)

In our series, 16 (64%) cases developed progressive obstruction on follow-up; nevertheless, obstruction was mild/moderate, requiring no intervention at the time of the last surveillance. Mild obstructive lesions were encountered at the initial diagnosis in two cases, with no intervention required, and slowly progressed to the moderate obstructive degree at a single level. Nine cases with initial COA/arch repair during the neonatal period slowly progressed to mild re-coarctation or were associated with mild to moderate mitral/aortic valvular stenosis or mild subaortic stenosis. Two patients had SAM resection, one with coarctation repair and the other as an isolated procedure, which later demonstrated reemergence of the membrane with minimal obstruction. Another case with a mildly stenotic parachute-like mitral valve and bicuspid aortic valve with tiny SAM underwent a transcatheter device occlusion of a residual PDA after surgical closure with only a moderate increase in the valvular stenosis. One patient with pulmonary hypertension resolved after a surgical operation at 15 months for mitral valve repair, SAM resection, and myomectomy had residual mild mitral regurgitation and mild stenosis, which progressed to moderate stenosis and regurgitation on the last follow-up at the age of 30 months. Lastly, another patient with pulmonary hypertension improved after aortic arch reconstruction with VSD and PDA closure in addition to mitral valve repair at five months of age, with only a newly developed mild mitral stenosis on the last surveillance at the age of 13 months. Regarding morbidity, 16% of cases required admission that was unrelated to elective surgery, and 28% of cases had persistent systemic hypertension after surgical correction. Mortality was encountered in 3 (12%), with two before scheduled intervention, one due to uncontrolled heart failure, and the other due to complications of prematurity in addition to heart failure.

**Fig. 4:**
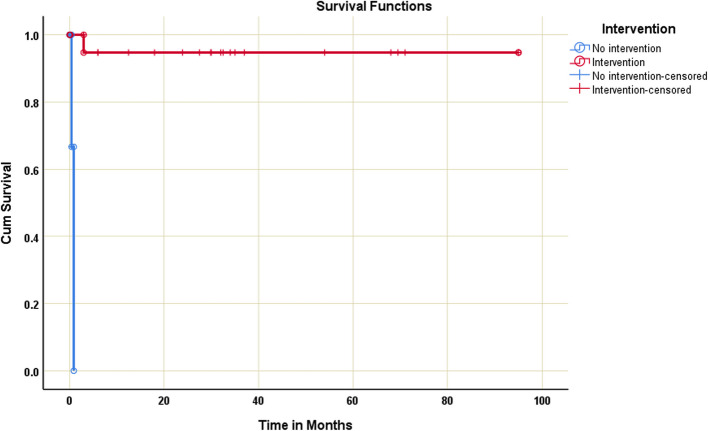
Cumulative Kaplan–Meier survival curves for SC patients with or without interventions (surgical/catheter). The p-value between patients'survival with or without interventions is < 0.001

Table 6 compares the demographics, anthropometrics, SC lesions, and interventions of surviving and deceased patients. Although the patient's weight was not significantly lower in the deceased group at presentation, the median Z-score did not differ. However, the patient's age, height, weight, and weight Z-score on the last follow-up were significantly lower in the deceased group. Such a pronounced difference seems expected due to the mortality in infancy for the three cases in the deceased category. Moreover, based on FS at the initial presentation, the proportion of cases with suppressed myocardial contractility was statistically significant in the deceased group (*p* = 0.03). Nevertheless, age at presentation, patient sex, lesions comprising the complex, presence of pulmonary hypertension, and percentage of cases that underwent interventions did not yield statistically significant differences between the two groups. However, a Kaplan–Meier survival analysis for patients who underwent intervention and those without intervention demonstrated a mean survival time for the intervention group of 90.2 months (SE = 4.7, 95% CI [80.9, 99.4]) and the non-intervention group 0.8 months (SE = 0.2, 95% CI [0.4, 1.1]) with log-rank test indicating a significant difference between the intervention and no-intervention survival distribution with a p-value of less than 0.001. Figure 4 illustrates the Kaplan–Meier survival curves.

**Table 6:** Comparison between alive and deceased patients regarding demographics, anthropometrics, SC components, and performed interventions

Variable		Alive* n=22	Died* n=3	*p*- value**
Demographics	Age at last follow-up (months)	34.5 (16.8–63)	0.9 (0.23–7)	0.02**
	Age at presentation(months)	1 (0.3–5.5)	0.9 (0.03–4)	0.5
	Sex (Male**)**	15 (68.2)	2 (66.7)	1
Anthropometrics	Wt at last follow-up (kg)	12.3 (8.9–16.1)	2.2 (1.7- 6)	0.02**
	Wt at presentation (kg)	4 (3.4–6.9)	2.2 (1.7–5.5)	0.2
	Wt Z-score at presentation	−1.4 (−2, −0.5)	−1.4 (−2.6, −0.2)	0.8
	Wt Z-score at last follow-up	−0.8 (−1.4, 0.1)	−2.4 (−2.6, −2.1)	0.04**
Components of SC	Supra-mitral membrane	3 (13.6)	1 (33.3)	0.4
	Parachute/Parachute-like mitral valve	18 (81.8)	3 (100)	1
	Bicuspid aortic valve	13 (59.1)	0	0.1
	Aortic coarctation	17 (77.3)	1 (33.3)	0.2
	Subaortic stenosis	12 (54.5)	1 (33.3)	0.6
Suppressed LV Contractility^$^		1 (9.1)	2 (66.7)	0.03**
Presence of PHT		3 (13.6)	1 (33.3)	0.4
Intervention		19 (86.4)	1 (33.3)	0.1

*Ht* height, *PHT* pulmonary hypertension, *SC* Shone’s complex, *Wt* weight

^$^Fractional shortening of less than 28% at the initial presentation

*Data are presented as median (interquartile range) or number (%)

**statistically significant (p-value ≤0.05)

## Discussion

Shone᾽s complex is one of the infrequently detected CHDs with limited prevalence. In the current work, we report a series of 25 cases of SC with a notable male predominance among the cohort, all of which were incomplete Shone subtype. Parachute mitral and COA were the most detected obstructive lesions, with non-invasive imaging sufficient for planning the required interventions. The mortality rate in our pediatric cohort with SC is 12%; all deaths occurred in infancy.

The median age of the current SC cohort at presentation was one month. This finding is compatible with Nicholson et al.'s results, who reported a median age of 28 days at presentation in a 20-year experience of pediatric SC [14]. These findings support the fact that most SC patients are mainly diagnosed during early infancy, possibly due to increased awareness about the complex, greater access to medical care, and the availability of echocardiography [15, 16]. Regarding the sex distribution, male predominance was observed in the current series, with 68% males. This observation aligns with several published series reporting on SC [14, 16, 17]. In contrast, Ma et al. reported that 53% of their participants were females [18]. However, there is no clear evidence that sex distribution affects the development of the complex.

All the studied patients in the current work had the atypical (incomplete) complex type, as per the classic Shone classification. On the contrary, Brown et al. reported typical SC in 7.4% of their series [15]. Additionally, Brauner et al. detected complete or typical SC in 10.5% of their cohort [19]. Li et al. found only two (3.0%) patients with the classic four obstructive levels [17]. However, atypical SC is far more prevalent than the complete form in most reports. Moreover, the newly suggested addition of LV obstructive lesions [4, 5] should be considered in further classifications for SC.

The most common obstructive anomalies in our SC series are PMV in 84%, COA in 72%, and SAM and BAV in 52% each. In accordance, Nicholson et al. reported that the most common lesion was COA (76.9%), followed by BAV (59.2%) [14]. According to the study by Li et al., the most common Shone-related anomaly was the annulo-leaflet mitral ring (75.8%) [17]. In our study, other associated cardiac anomalies were diagnosed in 44% of the SC cases. The two most common lesions are VSD and PDA, affecting 40% and 20% of patients, respectively. Other detected CHDs in association with SC include ASD, interrupted aortic arch, and aberrant right subclavian artery. Another association we found was the persistent left superior vena cava in 8% of associated anomalies. Consistent results were found by Li et al., with the highest associated lesions PDA (45.5%) and VSD (36.4%) [17]. In addition, Zhang et al. found that the two most commonly associated CHDs were VSD and PDA [20]. They also reported an aberrant right subclavian artery in one case and an interruption of the aortic arch in another [20]. Delmo Walter et al. found that the two most frequently associated lesions were PDA and ASD, followed by VSD with vascular ring in 6.7% of their cases [7].

Heart failure-related symptoms were the most common presentation in the current cohort, followed by subjective cyanosis. Similarly, a review of cases with SC in pregnancy proved that dyspnoea on exertion, orthopnoea, palpitations, and pulmonary edema were the presenting symptoms in 60% of the participants [21]. Shock could sometimes be the initial presentation [22]. Moreover, in the St Louis et al. series, 5 (17.9%) cases presented with cardiovascular collapse and 10 (35.7%) with heart failure symptoms [23]. On the contrary, heart failure was found in only one case (1.5%) of the Li et al. series [17]. Therefore, SC patients'clinical presentation and prognosis depend on the complexity and severity of the various obstructive lesions. Some cases could have a non-severe obstruction and present only with a murmur; others are diagnosed during investigation for failure to thrive, subjective cyanosis, recurrent chest infection, or hypertension, as in our current series. Despite the rarity, fetal diagnosis of SC is feasible using fetal echocardiography, with few reports of antenatal diagnosis [24–26]. In the current series, only one patient was diagnosed antenatally. Primary diagnosis of all cases was possible using TTE, with two cases requiring TEE to further delineate the mitral apparatus and supramitral area due to suboptimal transthoracic images. Kumar et al. reported five patients with SC who were evaluated using TEE [27]. In the current work, MSCT was required in 64% of patients to delineate the exact anatomy of COA and other associated anomalies in the aortic arch and its branches. However, none of our cases had CMR. Computed tomography and CMR are not routinely used in SC, although they may be beneficial in select cases to guide surgical or interventional approaches [17, 27–30]. Moreover, CT could be useful after multiple previous open-heart surgeries, where conventional echocardiographic assessment has limited resolution [31]. Due to this disease's complexity and individual variability, combining echocardiography with MSCT or CMR could help achieve a comprehensive diagnosis and design a structured management plan.

Diuretics were the most frequently prescribed medication category in our series, which is consistent with the high frequency of clinical heart failure in the currently described cohort. With severe arch hypoplasia, critical valvar aortic stenosis, or critical COA, urgent administration of prostaglandin E1 infusion is mandatory to maintain the PDA, which is essential to preserve systemic cardiac output.

The current series documented 4 cases of familial recurrence of CHD (16%). Most recurrences were detected among second-degree relatives, 2/4 (50%). The reported total familial recurrence rate of CHD among Egyptian families is 6.4% [32]. Furthermore, according to Fesslova's study, the total recurrence rate of CHD is about 4% [33]. Therefore, the SC constellation of lesions has a higher recurrence than CHD's reported total recurrence rate.

In 76% of our patients, surgical interventions were performed, and 16% had transcatheter interventions. The most common surgical procedure was COA repair, performed in 56% of patients with or without aortic arch reconstruction, followed by SAM resection in 16% of cases. The most common percutaneous intervention was balloon aortic valvuloplasty, accounting for 8%. Consistent results were demonstrated by Nicholson et al., who reported surgical interventions in 82% of patients, with 18% requiring catheterization. The most frequent initial surgical interventions in their series were COA repair and subaortic resection [14]. Similarly, Aslam et al. reported that 82% of patients underwent cardiac interventions during childhood. The most common initial interventions were COA repair, relief of LV outflow tract obstruction, and mitral valve repair or replacement [3]. However, Ma et al. reported a significantly lower prevalence of surgery in only 34% of cases, and 8% underwent catheter interventions [18]. Reintervention was performed in 12% of cases in the current series. In contrast, Brown et al. reported a higher reintervention rate in 66% of cases and a 31% reintervention rate in the Aslam et al. series [3, 15].

Regarding morbidity in our study, hospital admission unrelated to elective intervention was documented in 16% of cases due to decompensated heart failure and/or pneumonia. Persistent systemic hypertension after COA repair occurred in 28% of cases. One patient of our series required permanent pacemaker implantation and was admitted to the hospital due to arrhythmia and heart failure. A higher rate of hospitalization was reported by Aslam et al. in 48% of cases, primarily due to arrhythmias, heart failure, and infective endocarditis [3]. The mortality rate in the current study was 12%; two cases died in the NICU before intervention due to neonatal sepsis and heart failure, while another patient died after open heart surgery. The Nicholson et al. study reported a mortality rate of 14%, which is consistent with our findings. The median age at death in their series was 4.9 months. The 10-year survival rate for the entire cohort was 86.1%, with most deaths occurring within the first ten months of life [14]. On the contrary, Steele et al. reported no mortality until the age of 3 years [34]. St. Louis et al. reviewed 28 cases; two died after a second operative intervention, with an overall survival of 93% [23]. However, a higher mortality rate than our study was described by older reports such as Malhotra et al., who reported a mortality rate of 20.9%, with 5- and 10-year survival for staged surgical and transplantation was 88% versus 61.3% and 83.1% versus 61.3% [35]. Also, a high mortality rate was reported by Brauner et al., with an overall mortality rate of 26.5% and a 7-year survival rate of 73% [19]. The cause of variation in survival among centers is unclear, but it might be due to the differences in management approaches and the availability of surgical interventions.

The optimum management for cases with significant obstructive lesions is early correction before the onset of pulmonary hypertension. A good outcome is possible in SC, provided the early intervention [27, 36]. Poor outcome during surgical management is dependent on the degree of involvement of the mitral valve and the presence of secondary pulmonary hypertension [31]. However, Brauner et al. did not find an association between pulmonary hypertension and mortality, which contradicts the findings of the present study. Nevertheless, they noted a reversal in the degree of pulmonary hypertension in long-term survivors, which they partially attribute to improvements in perioperative care [19]. In the current study, there was no statistically significant difference between the survivors and deceased cases in the structural and interventional data; this may be due to the small sample size. However, significantly smaller weights and younger ages were noticed in the non-surviving group. Limited research has studied the risk factors impacting the outcome and reoperation of SC. Sugimoto et al. found that in congenital mitral valve disease, age of less than one year and being a part of SC were among the significant risk factors for death or reoperation [37].

One significant limitation of the current study was its single-center design and small sample size. Another drawback was the retrospective nature of the work, which imposed limitations on data collection, including some echocardiographic measurements. Multi-center studies involving larger sample sizes of SC patients should be conducted to confirm our results and facilitate further exploration of the risk factors influencing morbidity and mortality in SC patients. Moreover, further research is required to explore the potential of a genetic etiological basis for the complex.

## Conclusion

The variable constellation of multi-level left-sided obstructions in SC commonly presents as the atypical incomplete subtype. In the pediatric age group, the PMV and COA are the lesions most frequently encountered. A comprehensive evaluation of the complex can be achieved non-invasively through echocardiography and cardiac multi-slice computed tomography to guide the required interventions. Awareness about the potential course recurrence, progression, and reintervention of SC-related lesions should be conveyed to the family, emphasizing the need for a more tailored and prolonged follow-up of this disease. Furthermore, it is reasonable to recommend exploring the genetic etiologic background of the complex in future research.

## Data Availability

Relevant, de-identified data can be made available on reasonable request from the corresponding author.

## Abbreviations

CHD: Congenital Heart Disease
COA: Coarctation of the Aorta
CMR: Cardiac Magnetic Resonance
BAV: Bicuspid Aortic Valve
LV: Left Ventricle
MS: Mitral Stenosis
MSCT: Multi-slice Computed Tomography
PDA: Patent Ductus Arteriosus
PMV: Parachute Mitral Valve
SAM: Subaortic Membrane
SC: Shone᾽s Complex
TTE: Transthoracic Echocardiography
TEE: Transesophageal Echocardiography
VSD: Ventricular Septal Defect
